# Additively Manufactured Carbon Fibre PETG Composites: Effect of Print Parameters on Mechanical Properties

**DOI:** 10.3390/polym16233336

**Published:** 2024-11-28

**Authors:** Andreas L. Economides, Md Niamul Islam, Konstantinos P. Baxevanakis

**Affiliations:** Wolfson School of Mechanical, Electrical and Manufacturing Engineering, Loughborough University, Loughborough LE11 3TU, UKmdislam@lincoln.ac.uk (M.N.I.)

**Keywords:** additive manufacturing, viscoelastic characterisation, carbon fibres, quasi-static, tensile, compressive, composite material, dynamic mechanical analysis

## Abstract

This study investigates the quasi-static and viscoelastic properties of additively manufactured (AM) PETG reinforced with short carbon fibres. Samples were manufactured using different parameters in terms of the infill pattern, porosity, and annealing condition. Tensile and compressive tests were conducted to determine quasi-static properties such as Young’s modulus and toughness, and dynamic mechanical analysis was used under a frequency sweep of 1–100 Hz to describe the viscoelastic behaviour of the composites. The major impacts and responses between the print parameters were quantified using Analyses of Variance (ANOVAs), which revealed the major contributor to each mechanical property. Fractography on the tensile samples using scanning electron microscopy demonstrated fibre pull-out, indicating poor fibre–matrix bonding, but also revealed interfacial bonding between raster lines in the annealed samples. This had a prominent effect on the properties of latitudinal samples where the force applied was perpendicular to the raster lines. Generally, porosity appeared to have the greatest contribution to the variance in the mechanical properties, with the exception of the tensile modulus, where the infill pattern had a more substantial effect. Annealing caused a consistent increase in the tensile modulus of the tested samples, which can be used to support the design and optimisation of AM parts when they are used under specific loading conditions.

## 1. Introduction

Additive manufacturing (AM) is a key process for the rapid prototyping and development of products in modern industry. It is a product development method that diverges from traditional means of manufacturing such as injection moulding and CNC milling, as it consists of the addition rather than the removal of material from a manufactured part, which reduces the amount of waste and the overall cost, as well as the time required to make customised products [1]. Fused deposition modelling (FDM) continues to be the most popular method for producing AM structures due to its relatively low hardware costs compared to those of other AM processes (e.g., stereolithography, selective laser sintering), as well as the cost and variety of feedstock, which are higher in various other thermoplastic polymers. The most commonly found thermoplastics for AM are polylactic acid (PLA), acrylonitrile butadiene styrene (ABS), and polyamide (PA/Nylon). The feedstock is extruded through a heated nozzle, and the molten polymer is deposited onto a heated bed, where the extruded material binds layer by layer to the previously deposited material via solidification. AM polymer structures typically have a lower strength and stiffness than their bulk polymer counterparts, as they have microstructural defects such as voids and porosity [2,3,4]. Additionally, poor parameter selection and hardware setup can lead to defects such as delamination of the printed layers [5,6].

Rapid prototyping remains limited in its applications due to restrictions in build size and material variety, as well as production time [7]; however, developments in material engineering have introduced new feedstock into the market that features reinforcement in the form of fibres (carbon fibres, glass fibres, and nanocomposites) [5,8,9], with some studies exploring a plethora of natural, synthetic, organic, ceramic, and mineral fibres as possible reinforcing materials for AM [10].

The typical commercially available carbon-fibre-reinforced filaments for additive manufacturing comprise chopped/short fibres, which lead to anisotropic material properties. Furthermore, air pockets can be formed during AM due to the presence of moisture in the material, which, when passed through the extruder, is converted into steam and expands, forming voids that have detrimental effects on the mechanical properties of the printed part, such as reductions in its strength and stiffness [11]. A recent study also explored the in-house manufacture of reinforced PETG using carbon black as a particulate reinforcement by incorporating carbon black nanoparticles into a commercially available PETG matrix via melt mixing [12]. However, this study focused on the mechanical properties of commercially available carbon-fibre-reinforced PETG without making any in-house modifications to the material, as most businesses and institutions do not have filament manufacturing capabilities and would most likely opt for a commercially available solution when printing with reinforced feedstock. The matrix performs several roles, such as preventing buckling of the fibres under compressive loads, transferring the load to the fibres, and protecting the fibres from mechanical damage. The performance of the composite, however, is heavily dependent on the adhesion between the fibre and the matrix, which is affected by the type and quality of the bond formed [13]. PETG (Polyethylene Terephthalate, Glycol-Modified) is an ideal matrix material due to its ease of use in AM. It is classified as the middle ground between PLA and ABS, as it does not warp very easily, and it is generally considered to have good bed adhesion [14].

Annealing is an additional post-processing method for improving the mechanical properties of AM components, as it is mostly used to increase the crystallinity of a semi-crystalline polymer. Additionally, annealing serves as a post-processing technique to further increase the interfacial bonding between individual printed raster lines. Bond formation takes place, allowing raster lines which are in contact to form a neck and polymer molecules to diffuse, which, in turn, forms an interfacial bonding zone [15,16,17,18]. Studies have also shown that annealing releases residual thermal stresses via viscoelastic relaxation of the polymer [19]. Annealing is achieved by heating an AM component to a specified temperature, typically above the material’s glass transition temperature, holding the temperature for a specified amount of time, and gradually cooling the component back to room temperature.

The current research on AM carbon-fibre-reinforced PETG, denoted herein as CFRPETG, structures lacks an in-depth analysis of their quasi-static performance and viscoelastic properties under a frequency sweep, as these studies have only focused on bending and thermal conductivity [20] or tensile testing [21,22], without exploring the effects of varying the infill pattern, porosity, or post-processing conditions on the manufactured components, nor the compressive performance of CFRPETG. This paper aims to fill this gap and compare the commonly tested primary mechanical properties found in the literature (elastic modulus, ultimate tensile strength, etc.) by thoroughly investigating the quasi-static and viscoelastic performance and microstructure of AM CFRPETG using quasi-static and dynamic experimental testing methods. The primary focus was on the tension–compression properties, i.e., strength, stiffness, and toughness, in response to different manufacturing factors, such as infill percentage and pattern, with detailed statistical analysis, including the effect of annealing, along with fractography and failure mode analysis. Additionally, a DMA under a frequency sweep was conducted rather than a thermal analysis, as this was already found in the existing literature, as well as because PETG is an amorphous polymer.

Similar to the study by García et al. [23], the influence of each manufacturing parameter (infill, porosity, and post-processing) was explored using ANOVA to determine the contribution of each parameter to the mechanical properties of the AM structures and to identify any significant interactions between each of the parameters. Understanding the influence of each parameter lays the foundation for manufacturing optimisation, such that functional prototypes perform to the specification while taking factors such as time, material availability, wastage, cost, and print orientation into consideration. By exploring the effects of porosity under different infill patterns and post-processing conditions, the material usage can be reduced while also obtaining similar mechanical properties to those of solid structures, thus contributing to the aforementioned factors.

## 2. Methodology

### 2.1. Materials and Manufacturing Methods

**Figure 1:**
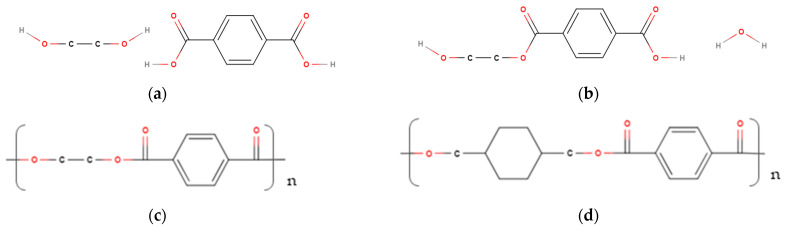
(**a**) PET constituent monomers; (**b**) PET monomer; (**c**) PET repeating unit; and (**d**) PETG repeating unit.

The current study featured the use of material-extruded additively manufactured (MEAM) PETG polymer–matrix composites reinforced with short carbon fibres. The specimens were tested with different infill percentages and filament orientations, along with the effects of further post-processing conditions (annealing). PET (Polyethylene Terephthalate) polymer chains consist of terephthalic acid and ethylene glycol, which react via condensation polymerisation (esterification) to form ethylene terephthalate and react again immediately via polycondensation to produce PET. In contrast, PETG replaces glycol with cyclohexanedimethanol (CHDM) as the starting monomer, which introduces an additional 6 carbon atoms that disrupt the secondary bonds between the polymer chains (Figure 1). This results in a softer, amorphous polymer with a higher shock resistance, a lower melting temperature, and a higher glass transition temperature [24].

**Figure 2:**
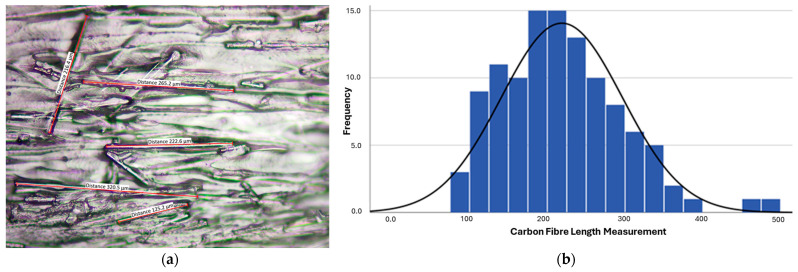
(**a**) Carbon fibre length measurements using optical microscopy; (**b**) histogram of carbon fibre length.

The CFRPETG feedstock was obtained through RS Components (Carbon-P filament), consisting of a 20%-fibre-volume fraction of chopped carbon fibres with an average length of 210 μm, as determined using optical microscopy (Figure 2a). A total of 110 measurements (Figure 2b) were made to estimate the median fibre length and to eliminate outliers (partially visible fibres, broken segments, etc.). Also, SEM was used to determine the fibre diameter to be 60 μm.

To determine the reinforcement properties of a fibre within a matrix, the average fibre length must be compared to the critical fibre length. Fibres with a length less than the critical fibre length provide reinforcement similar to particulates, fibres with lengths greater than $l_{c}$ and less than 15$l_{c}$ have intermediate fibre reinforcement properties, and fibres with lengths greater than 15$l_{c}$ exhibit continuous fibre reinforcement properties [25]. Further, the critical length formula was used to describe the behaviour of the fibres within a matrix:

(1) $$l_{c}=\frac{\sigma_{f}D}{2\tau },$$

where $l_{c}$ is the critical fibre length, $\sigma_{f}$ the fibre strength, D the fibre diameter, and τ the interfacial shear strength. Wu et al. obtained the interfacial properties of carbon fibres and polyamide where the shear strength of the two materials was close to 30 MPa [26]. No sources characterising the interfacial shear strength of PETG and carbon fibres were identified; hence, the value for polyamide was used, as it was the most comparable material available. Additionally, a fibre strength ($\sigma_{f}$) of 7.82 GPa was used [27].

The resulting critical fibre length calculated was 804 μm, which was significantly greater compared to the median carbon fibre length obtained using microscopy (210 μm); therefore, the carbon fibres in the matrix were determined to act as particulates rather than fibres [25].

Cura software version 5.9.0 and an Ultimaker 2+ FDM 3D printer (Ultimaker, Zaltbommel, The Netherlands) were used to manufacture the CFRPETG specimens. The printing parameters used throughout the manufacturing process are summarised in Table 1. It is worth noting that the layer height and the extrusion width were kept the same throughout the manufacturing process such that the distribution of the reinforcement remained mostly unchanged and had a minimal effect on the mechanical properties of the samples [28,29].

**Figure 3:**
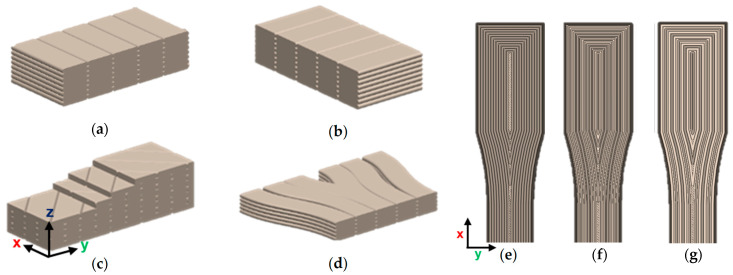
Cura infill patterns used relative to the World Coordinate System (WCS) and visualisation of infill percentages in concentric infill pattern: (**a**) longitudinal; (**b**) latitudinal; (**c**) cross-ply; (**d**) concentric; (**e**) 100% infill; (**f**) 80% infill; and (**g**) 40% infill.

The variables that were controlled in the investigation were the infill percentage, the infill pattern, and annealing (Figure 3). The infill percentage reflects the material deposited within the internal structure of the specimen, the infill pattern indicates the orientation in which raster lines are deposited, and the annealing condition indicates whether or not the sample was heat-treated or not (the annealing process is outlined below). Combinations of 4 infill patterns (cross-ply, concentric, latitudinal, and longitudinal), 3 infill percentages (40%, 80%, and 100%), and 2 annealing conditions (annealed or as-printed) were selected for testing, which resulted in 24 combinations. The test samples were kept in an airtight plastic bag alongside silica gel packets to mitigate any effects on the specimens’ mechanical properties due to moisture absorption because of the material’s hygroscopic nature [30,31,32].

Specimens were annealed using a heat treatment kiln at 85 °C for 30 min: the samples were placed in a cold kiln, brought up to temperature, heat-treated for 30 min, and left to cool gradually inside the kiln so that warping was minimised. Compared to Dupaix and Boyce’s study [33], the heat treatment time used in this study was reduced by half. This was carried out to analyse the effect of reducing the treatment time on the material’s mechanical properties, which is linked to energy consumption and the post-processing time.

### 2.2. The Mechanical Test and Microstructural Analysis

**Figure 4:**
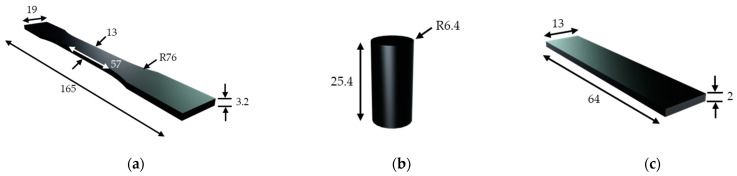
Testing specimen specification and dimensions (in mm): (**a**) tensile; (**b**) compressive; and (**c**) DMA.

Tensile tests were conducted following the ASTM D638-14 standards, using Type I specimens at a speed of 5 mm min^−1^ until fracture (Figure 4a) [34]. Similarly, compression testing was performed in adherence to the ASTM D695-15 standards at a speed of 1 mm min^−1^ using cylindrical specimens (Figure 4b) [35]. During the tensile tests, stresses were applied on the x-axis; compressive testing was performed on the x-axis for longitudinal samples and on the z-axis for all other samples, and the DMA specimens were tested on the z-axis. At least three specimens of each type were tested to ensure the precision of the test results, so overall, 72 specimens were manufactured for each tensile and compressive test.

Dynamic mechanical analysis (DMA) testing was carried out according to the ASTM D5023-15 standards in a three-point bending configuration (Figure 4c) [36] to determine the viscoelastic properties of the AM composites. A frequency sweep of 1–100 Hz at 5 Hz intervals was used at a constant temperature of 22 °C. An amplitude of 0.5 N oscillation and a preload force of 12 N were applied to ensure that the specimens maintained contact with the vibrating shaft throughout a complete oscillation. No distinct temperature difference was recorded throughout the testing procedure, with three repetitions completed for each specimen type. Due to material limitations, only longitudinal and latitudinal infill patterns were tested, with two specimens of each type manufactured for a total of four samples for the DMA.

Prony series can be used to describe the viscous behaviour of materials. In the time domain, the dimensionless shear moduli is provided as

(2) $$G_{R}\left(t\right)=1-\sum_{i=1}^{N}G_{i}\left(1-e^{-\frac{t}{\tau_{i}}}\right),$$

where $G_{R}$ is the dimensionless shear modulus, t is time, and $G_{i}$, $\tau_{i}$ for i=1,2,…,N are the material constants [37,38].

The Fourier transform is applied to Equation (2) to present the storage and the loss moduli in the frequency domain as

(3) $$G_{s}\left(\omega \right)=G_{O}\left(1-\sum_{i=1}^{N}G_{i}\right)+G_{0}\sum_{i=0}^{N}\frac{G_{i}\tau_{i}^{2}\omega^{2}}{1+\tau_{i}^{2}\omega^{2}},$$

(4) $$G_{l}\left(\omega \right)=G_{0}\sum_{i=0}^{N}\frac{G_{i}\tau_{i}\omega }{1+\tau_{i}^{2}\omega^{2}},$$

where $G_{s}$ and $G_{l}$ are the storage and loss moduli, respectively; $G_{0}$ is the instantaneous shear modulus; and ω is the frequency.

The instantaneous shear modulus, $G_{0}$, can be approximated as

(5) $$G_{0}=\frac{E}{2\left(1+v\right)},$$

where E is the elastic modulus and v is Poisson’s ratio, estimated as equal to 0.3.

A generalised reduced gradient nonlinear solver was used to estimate the Prony series parameters $G_{i}$ and $\tau_{i}$ which were optimised for the corresponding number of terms N by minimising the squared residuals between the experimental values of the storage and loss moduli, as well as $G_{s}$ and $G_{l}$, via calculation through the Prony series parameters.

## 3. Results

### 3.1. Tensile Testing

**Figure 5:**
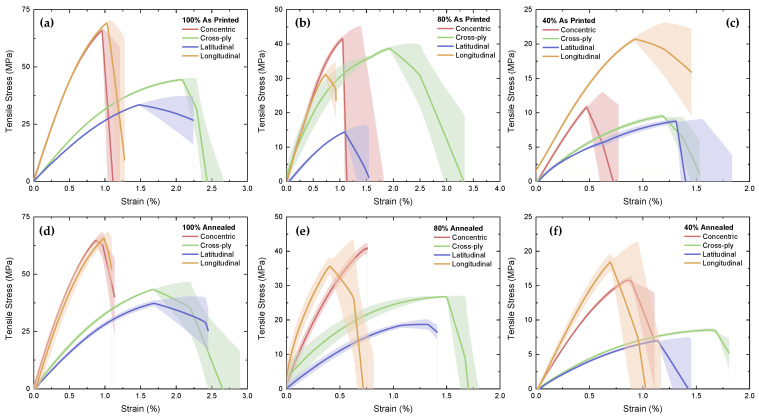
Mean stress against strain of CFRPETG samples from tensile tests (shaded region based on error bars) for: (**a**) 100%; (**b**) 80%; (**c**) 40% as printed samples; and (**d**) 100%; (**e**) 80%; (**f**) 40% annealed samples.

The mean outputs of the tensile tests of the MEAM CFRPETG specimens are shown in Figure 5. Overall, the longitudinal samples had a higher ultimate tensile strength (UTS) and a higher Young’s modulus than the specimens with the other infill patterns, with the only exceptions being the 80%-infill samples, where for both the as-printed and annealed cases, the concentric samples exhibited a greater UTS. The latitudinal samples, on the other hand, consistently displayed the lowest UTS and Young’s modulus. Comparing the as-printed and annealed cases, the 80%-infill samples (Figure 5b,e) displayed a lower tensile strain, as the annealed concentric and longitudinal samples fractured within the 0 to 0.75% strain regime, whereas their as-printed counterparts fractured beyond 1% strain. However, the cross-ply samples revealed more ductile behaviour when annealed at 40% infill (Figure 5f), fracturing at close to 1.7% strain rather than the 1.2% found in the as-printed tests.

#### 3.1.1. Average Ultimate Tensile Strength (UTS)

**Figure 6:**
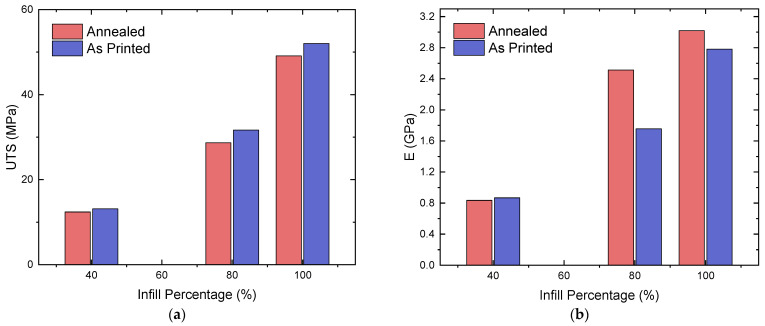
Average ultimate tensile strength (**a**) and tensile moduli (**b**) of annealed and as-printed specimens.

For all of the infill percentages, the sample with the highest UTS was the as-printed sample, unlike findings from similar studies, such as Kumar et al. [22], where annealed samples had higher values than their as-printed counterparts. In order of increasing percentage infill, the highest values for UTS were 23.0 (longitudinal), 43.7 (concentric), and 67.8 MPa (longitudinal), respectively. The highest value of UTS was 67.8 MPa for the 100%-infill, as-printed, longitudinal sample, whereas its annealed counterpart had a value of 59.4 MPa, a decrease of 12.3%. The UTS improved significantly for the annealed 80%-infill longitudinal and latitudinal samples, by 24.5% (30.4 to 37.8 MPa) and 19.8% (14.3 to 17.1 MPa), respectively (Table 2). Out of 12 averages, only 4 annealed samples experienced an increase in the UTS. Evaluating comparable specimen types from Kumar et al. (80%-infill, as-printed, longitudinal: 29.5 MPa) [39] and Kumar et al. (100%-infill, annealed, longitudinal: 70.3 MPa) [22], the corresponding UTS values from this study were 30.4 MPa and 59.4 MPa, respectively. The 80%-infill specimens seemed to conform with each other; however, the 100%-infill annealed samples had a 10.9 MPa difference between them, suggesting high sensitivity in the data collected. These differences result from the large number of manufacturing parameters associated with the MEAM process. The average UTS output for each infill density before and after annealing is presented in Figure 6a. Comparing the UTS of the longitudinal samples to that observed in a study using samples with particulate reinforcement printed in a similar orientation, a value of 67.8 MPa is reported herein, in comparison to the maximum value of approximately 43 MPa at a 1 wt% carbon black concentration in [12].

#### 3.1.2. Average Tensile Modulus

The tensile modulus consistently increased throughout all of the annealed samples compared to the average UTS values, with only 3 out of 12 averages being lower after annealing, resulting in a higher average tensile modulus for the annealed specimens compared to that of the as-printed parts (Figure 6b). The annealed longitudinal samples had the highest values across the different infill percentages, with average values of 4.58, 4.13, and 1.37 GPa in order of decreasing infill percentage. Evaluating the difference between the annealed and as-printed samples, the greatest percentage increase in the tensile modulus was experienced by the 80%-infill samples, where the concentric, latitudinal, and longitudinal values increased by 40.8%, 34.9%, and 73.5%, respectively. The samples which did not show an increase in their tensile moduli were the 40%-infill cross-ply concentric and latitudinal samples. Compared to similar studies by Kannan et al. (100%-infill, cross-ply, non-annealed: 2.34 GPa) [21], the equivalent value from this study was 1.63 GPa, with a difference of 0.71 GPa between these two values. This variance can be justified by the different machine parameters used in said investigation (a 0.4 mm nozzle diameter and a 0.15 mm layer height).

#### 3.1.3. Average Tensile Toughness

**Figure 7:**
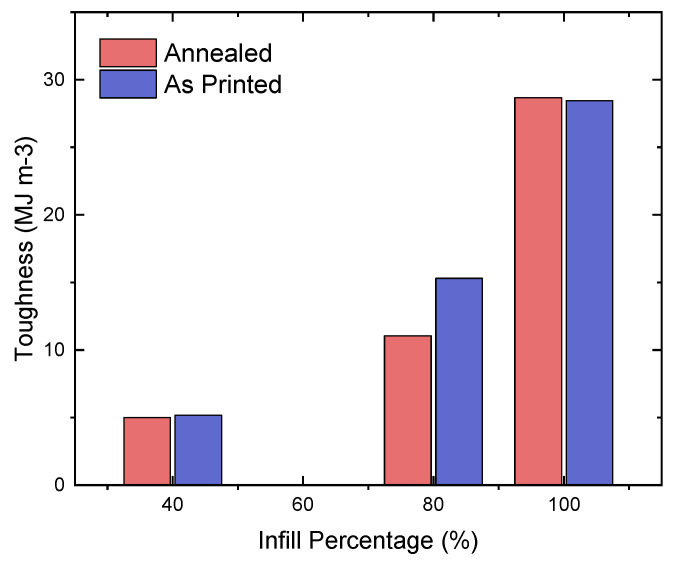
Average toughness of annealed and as-printed specimens.

Similar to the values for the UTS, annealing had an irregular effect on improving the toughness of the samples, where 7 out of 12 averages were higher for the annealed compared to the as-printed samples. Table 2 reveals that the annealed cross-ply samples had the highest toughness at 100% infill, at 35.8 $MJ\;m^{-3}$; the as-printed cross-ply samples had the highest toughness at 80% infill, at 33.0 $MJ\;m^{-3}$; and the as-printed longitudinal samples had the highest toughness at 40% infill, at 8.5 $MJ\;m^{-3}$. The greatest increase in toughness was exhibited by the 40%-infill concentric sample, which had an increase of 164.4% in toughness, from 2.0 to 5.2 $MJ\;m^{-3}$. Comparably to the UTS, at 80% and 100% infill percentages, the latitudinal samples showed a significant increase in their toughness values (of 31.5% and 29.0%, respectively). The average response at each infill density before and after annealing is presented in Figure 7.

#### 3.1.4. ANOVA of the Tensile Test Results

All factors were statistically significant (p<0.05) in the ANOVA of the UTS, with the infill percentage having the greatest contribution (69.25%), followed by the infill pattern (21.14%) and the annealing treatment (0.35%) (Table 3). Hence, it can be concluded that the primary factor affecting the UTS of a sample is the infill percentage. Further, the only variables which do not show a significant interaction are the infill percentage and annealing (Infill Percentage × Annealing, p>0.05), indicating that the effect of annealing is not as pronounced for specific types of infill percentages.

Similarly, all factors can be considered significant for the tensile modulus (p<0.05), with the infill pattern having the greatest contribution (43.15%), followed by the infill percentage (39.87%) and annealing (1.43%) (Table 4). Comparing the contributions to the UTS and the Young’s modulus, annealing causes an increase of 1.08%, the infill pattern becomes the primary contributor (at an increase of 22.01%), and the infill percentage becomes the secondary contributor (at a decrease of 29.38%). Based on this analysis, the primary factor affecting the tensile modulus of a sample is the infill pattern, and the infill percentage is almost equally important. All variables appear to have a significant interaction between them (p<0.05), indicating that the effect of changing the infill pattern or the annealing for different infill percentages yields a stronger effect. The results also suggest that it is more beneficial to use an 80%-infill longitudinal sample (2.38 GPa) than a 100%-infill, cross-ply sample (1.63 GPa), as less material can be used but a greater tensile modulus is yielded.

**Figure 8:**
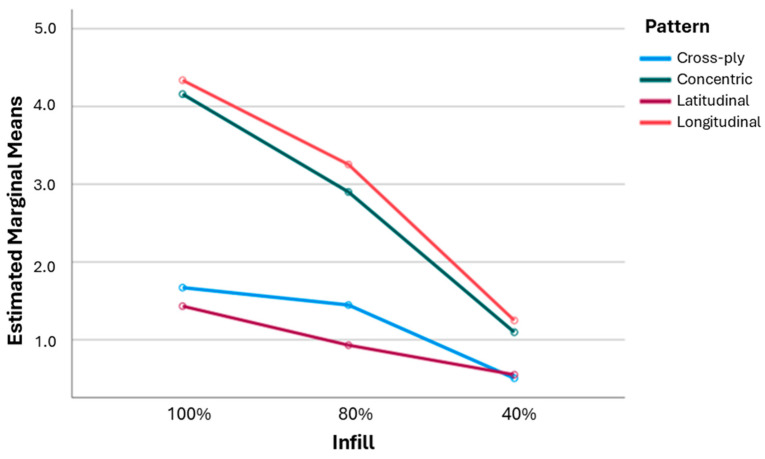
Interaction effect diagram for tensile modulus.

The only variables which appear to have a significant interaction are the infill percentage and pattern (Infill Percentage × Infill Pattern, p<0.05), where a change in the infill pattern yields stronger effects for specific infill percentages. The interaction effects are more apparent when they are represented graphically, as strong and weak interaction effects are more distinguishable (Figure 8). A lower infill % (increased porosity) decreased the overall performance of the samples, where the effect was greater for specific infill patterns. The effect of a decreasing infill percentage had a greater effect on the tensile moduli of longitudinal and concentric samples compared to those of cross-ply and latitudinal samples.

Annealing does not have a significant effect (p=0.330) on toughness compared to the mechanical properties previously analysed (Table 5), whereas the infill percentage has the greatest contribution (61.26%), followed by the infill pattern (10.29%). It is also worth noting that there is a sizable increase in the contribution of the error to the variance (15.68%) compared to the error in the UTS (1.14%) and the Young’s modulus (1.35%).

#### 3.1.5. Analysis of the Tensile Failure Mode 

**Figure 9:**
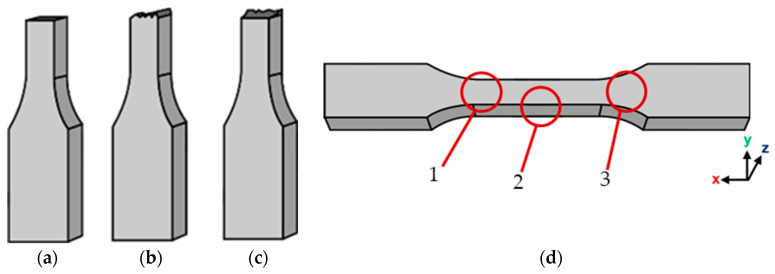
(**a**) Clean and flat failure site; (**b**) clean and irregular failure site; (**c**) rough and highly irregular failure site; and (**d**) failure locations observed during testing.

Failure types can be characterised by the shape, location, and fracture type at the failure site (Figure 9). A “clean” failure site can be described as a flat fractured surface with no significant irregularities, as presented in Figure 9a. Accordingly, Figure 9b shows a relatively clean but irregular failure site, where the surface is slightly rough due to shear failure, and Figure 9c a highly irregular failure site, where the surface is very rough, and there is an uneven breakage of the specimens. The failure locations observed during testing were (1) within the gauge length but very close to the fillet radius, (2) within the gauge length, and (3) outside the gauge length in the fillet area (Figure 9d).

The failure mode for each specimen type tested is summarised in Table 6. There was an evident trend between the infill percentage and failure type, as specimens with a lower infill percentage were more likely to experience type (b) and type (c) failure types. Additionally, only the annealed, 40%-infill samples experienced a type (b) failure type, as opposed to the 40%-infill, as-printed samples, which may have contributed to their different failure mechanisms. It is also worth noting that this applied to both longitudinal and concentric samples—two very similar pattern types, where the force applied is parallel to the direction of the raster lines.

**Figure 10:**
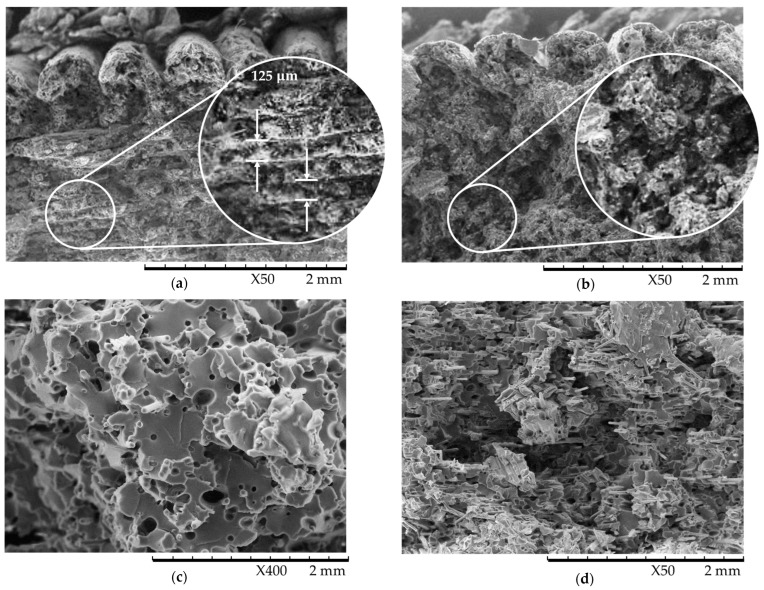
Fractography of as-printed latitudinal sample (**a**), annealed longitudinal sample (**b**), as-printed latitudinal sample (**c**), and annealed cross-ply sample (**d**).

The fractographic examination of both the annealed and as-printed samples is summarised in Figure 10. The raster layers of the as-printed sample are clearly defined and visible on the failure surface (Figure 10a), whereas on the annealed samples (Figure 10b,d), there is no discernible difference between the raster layers. By annealing the material, a bond formation mechanism is permitted, by which two raster lines in contact form a neck and begin to diffuse polymer molecules to form an interfacial zone. This interfacial zone allows for the formation of additional secondary bonds between molecules from different raster layers, which, in turn, increases the part’s mechanical properties [40]. This process has a more prominent effect on the mechanical properties of the latitudinal samples, where the force is perpendicular to the raster lines. Based on the findings from the ANOVAs, annealing consistently had a greater effect on the material’s tensile modulus compared to its UTS and toughness, apart from for the latitudinal samples.

Exploring the fibre–matrix adhesion in the samples using fractography, it is apparent that there is a significantly higher percentage of fibre pull-out in the as-printed samples (Figure 10c) than in the annealed samples (Figure 10d), evident in the higher number of voids/holes left by fibre pull-out. This indicates that annealing may have a positive effect on the fibre–matrix adhesion of tensile samples, which is reflected in their more brittle behaviour.

### 3.2. Compressive Testing

**Figure 11:**
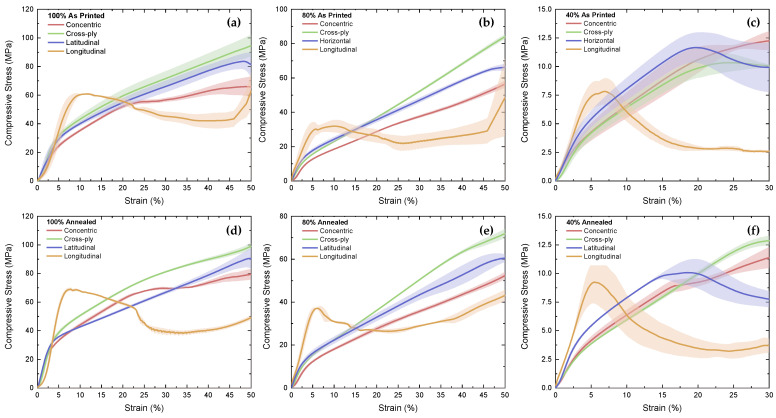
Mean stress against strain of CFRPETG samples from compressive tests (shaded region based on error bars) for: (**a**) 100%; (**b**) 80%; (**c**) 40% as printed samples; and (**d**) 100%; (**e**) 80%; (**f**) 40% annealed samples.

The mean outputs of the compressive tests for the different MEAM CFRPETG samples are presented in Figure 11. Similar to the results of the tensile tests, the longitudinal samples had a consistently higher compressive modulus at the same infill percentage and with the same post-processing type. The cross-ply specimens regularly showed the greatest compressive strength beyond the 15% strain regime, with the exception of the 40%-infill samples. Annealed samples appeared to perform in a more repeatable manner than their as-printed counterparts, as evidenced by the spread of the results. The 40%-infill, as-printed samples (Figure 11c) had the greatest spread in the measurements, evident by the area of the error bars, due to the slight variations in the distribution of the material throughout the printed structures.

#### 3.2.1. Average Compressive Yield Strength

**Figure 12:**
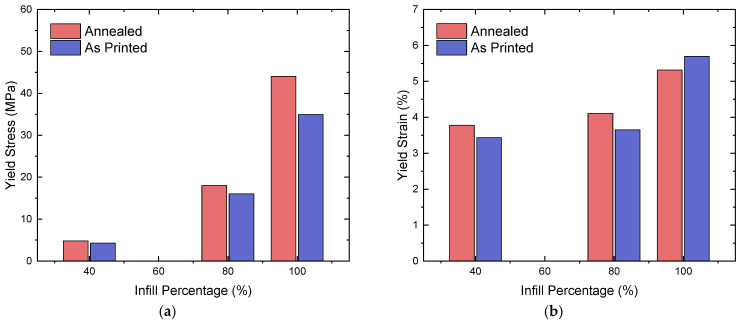
Average yield stress (**a**) and yield strain (**b**) of annealed and as-printed specimens.

Compared to the tensile testing, annealing showed much more consistent results in terms of improving the strength of the tested samples. Out of 12 sample types, only 3 exhibited a decrease in yield strength post-annealing; however, they were all not 100%-infill specimens and did not show a decrease in strength of greater than 10%. The annealed longitudinal sample is consistently the strongest in its infill percentage category and is closely followed by its as-printed counterpart (Table 7). The greatest yield strength values in decreasing order of infill percentage are 68.7, 37.1, and 9.2 MPa for the longitudinal, annealed samples. The yield stress appears to show an exponential trend, as the values before and after annealing are almost equal at a 40% infill and increase nonlinearly throughout 80% and 100% (Figure 12a). Similarly, the variance between samples with different infill patterns and annealing increases as the infill percentage increases. Comparing the annealed and as-printed samples, there is an increase in yield strength for all of the annealed, longitudinal samples (Table 7). The percentage increase in strength becomes greater as the infill percentage increases—11.8%, 12.2%, and 26.0%, respectively.

#### 3.2.2. Average Compressive Yield Strain

The highest values for compressive yield strain in order of decreasing infill percentage belong to the longitudinal samples; however, the as-printed sample with 100% infill has a higher value compared to its lower infill counterparts—with 8.97%, 6.06%, and 5.56% strain, respectively (Table 7). There is no significant trend for yield strain against infill percentage, as certain 40%-infill samples experience a greater yield strain than their 80%-infill equivalents. However, annealing appears to produce relatively consistent results between infill percentages and patterns, as only 4 out of 12 sample types decreased in their values when they were annealed. Interestingly, concentric patterns showed consistent improvements: as the infill percentage decreased, the effect of annealing became greater, with 10%, 27%, and 64% increases in the yield strain values in decreasing order of infill percentage. A similar trend can be observed between the 80%- and 40%-infill longitudinal samples, where there are 8% and 13% increases in yield strain, respectively (Figure 12b). The 100% longitudinal samples were an exception to this trend, as the annealed samples experienced an 18% decrease in their average yield strain. The average response for each infill density before and after annealing is illustrated in Figure 12b.

#### 3.2.3. Average Compressive Modulus

**Figure 13:**
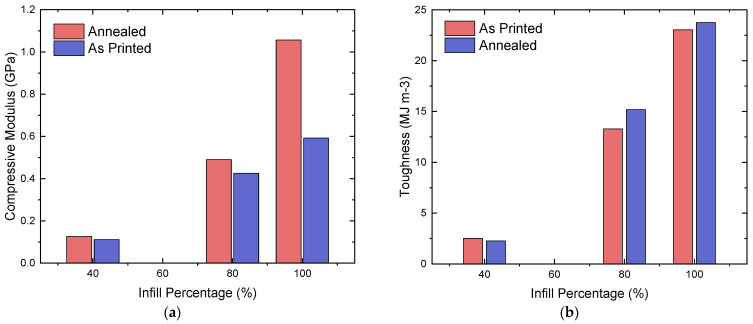
Average compressive modulus (**a**) and toughness (**b**) of annealed and as-printed specimens.

Similar to the yield strength of the compressive specimens, the compressive modulus also appears to exhibit an exponential trend with respect to the infill percentage. In decreasing infill percentage order, the highest compressive modulus values are 1.65, 0.77, and 0.19 GPa, where these values belong to the annealed, longitudinal samples. The exponential trend is more apparent for the annealed longitudinal and cross-ply samples, where their values increase significantly between 80% and 100% infill. In terms of the consistency of the effect of annealing, only 2 out of 12 sample types demonstrated a decrease in their compressive modulus when annealed—40%-infill and 80%-infill concentric samples. The consistency of the effect of annealing on the compressive modulus can be observed in Figure 13a, where in decreasing infill percentage order, the compressive modulus increased by 107%, 24%, and 48%.

#### 3.2.4. Average Compressive Toughness

In contrast to the other mechanical properties, compressive toughness appears to exhibit a more linear trend across different infill densities (Figure 13b). The highest value at 100% infill belongs to the annealed cross-ply sample and to the as-printed cross-ply samples at the other infill percentages. The corresponding values are 34.78, 22.51, and 6.58 $MJ\;m^{-3}$ in decreasing order of infill percentage (Table 7). Only 5 of the 12 sample types showed an increase in their compressive toughness when annealed, which indicates that annealing does not consistently improve this property across different infill patterns. It is worth noting that all of the infill patterns showed a slight increase in toughness at 100% infill, and the longitudinal samples experienced a 3%, 14%, and −10% change in their toughness in decreasing infill percentage order when annealed.

#### 3.2.5. ANOVA of Compressive Test Results

The infill percentage has the greatest contribution (57.86%) to the variance in the yield strength of the compressive samples, followed by the infill pattern (25.97%) and annealing (0.74%), all of which are significant (p<0.05) (Table 8). The only significant interaction between factors is that between the infill percentage and the pattern, and it contributes to approximately 10.87% of the variance in yield strength. It is also worth noting that error contributes only 3.32% to the ANOVA.

Unlike yield strength, not all of the parameters are significant in terms of their contribution to the variance in yield strain. Namely, annealing has a *p*-value of 0.214, which makes it a non-significant factor. The infill percentage contributes to 5.46% and the pattern to 57.41% of the variance in yield strain (Table 9). Similar to yield stress, the only significant interaction is that between the infill percentage and pattern, which contributes to 8.60% of the variance. For this ANOVA, the error is much greater (24.54%), which is an indication to interpret the data with caution.

Proceeding to the ANOVA for the compressive modulus, all three variables are significant—infill percentage contributes 70.78%, infill pattern 9.88%, and annealing 2.00% to the variance in the data (Table 10). The interaction between infill percentage and pattern, as well as that between infill percentage and annealing, is significant, with each contributing 3.63% and 4.94% to the variance, respectively. Comparing the contribution of annealing to the compressive modulus in contrast to that of the other mechanical properties, it is evident that it is much more significantly quantified by the values in Table 7. Furthermore, the error in the ANOVA is minimal (6.56%), making the values of the contributions to the compressive modulus more reliable. 

Infill percentage contributes to most of the variance in compressive toughness (91.55%), followed by the infill pattern (4.49%) and annealing (0.16%), all of which are significant (Table 11). There are significant interactions between the infill percentage and the pattern, as well as the infill percentage and annealing, with each contributing 1.79% and 0.44% to the variance, respectively. Moreover, the error in the ANOVA is insignificant (1.16%).

#### 3.2.6. Analysis of the Compressive Failure Mode 

**Figure 14:**
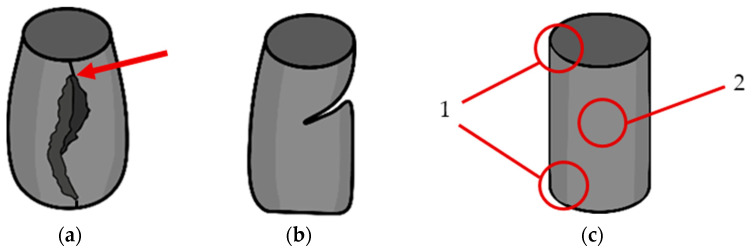
(**a**) Barrelling and crack formation (red arrow highlights the location of the z-seam); (**b**) buckling and interlaminar failure; and (**c**) failure locations observed during testing.

**Figure 15:**
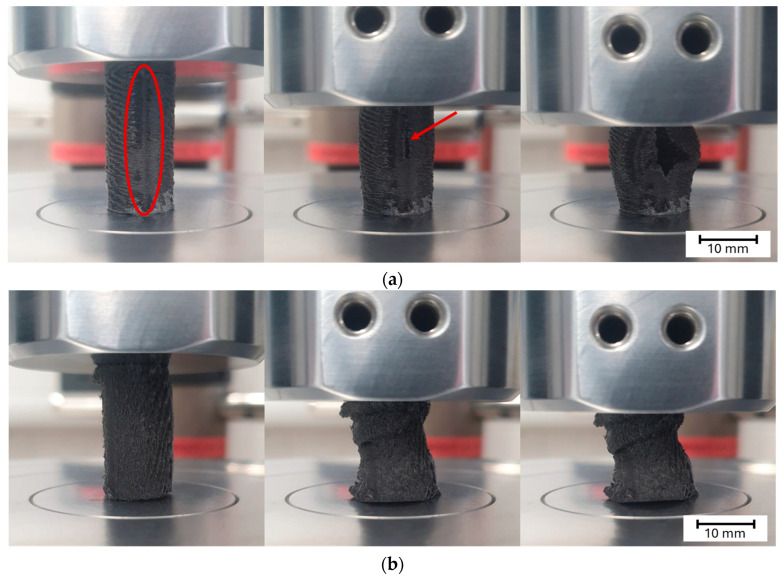
(**a**) Barrelling and crack formation progression (red circle highlights z-seam, red arrow highlights crack formation at the z-seam); (**b**) buckling and interlaminar failure progression.

The samples tested under compression appeared to mainly experience two types of failure. Figure 14a shows a combination of barrelling and crack formation and propagation along the side of the sample caused by shear failures, and Figure 14b a combination of buckling and interlaminar failures. In terms of the failure locations (Figure 14c), failure was mainly observed at the top and bottom of the specimens (1), as well as alongside the middle of the specimen’s length (2). It is worth noting that for the majority of the samples that failed at location (2), crack formation and propagation occurred along the specimen’s z-seam (Figure 14a and Figure 15a). It was observed that in most of the samples tested, regardless of the type of failure experienced, cracks formed and propagated at the z-seam, similar to in [41]. The z-seam is the visible line along the height of a printed part caused by unwanted extruded material in the course of the z-jump of the nozzle during a layer change [42].

All of the cross-ply, concentric, and latitudinal samples experienced a type (a) failure, with most of the samples beginning to fail at location (2) (Figure 15b). Some of the samples began to shear at location (1); however, their number was insignificant enough to be considered an anomaly. The longitudinal samples experienced a type (b) failure, with all of the samples beginning to buckle before showing any major deformation. The samples buckled at location (2); however, a large number of samples began to experience a combination of interlaminar and shear failure at location (1).

### 3.3. DMA Testing

**Figure 16:**
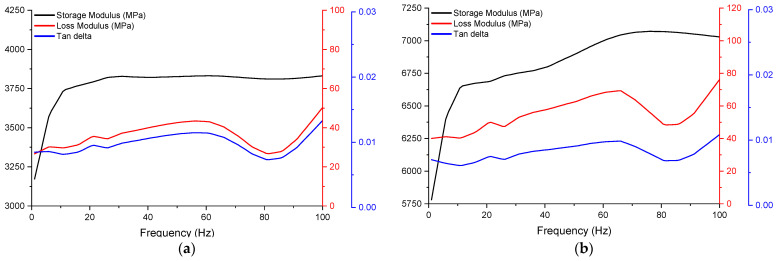
Mean storage modulus, loss modulus, and tan delta of latitudinal (**a**) and longitudinal samples (**b**).

A total of four DMA samples were tested, printed with 100% infill and in longitudinal and latitudinal infill patterns. The mean storage modulus, loss modulus, and tan delta using a frequency sweep of 0–100 Hz at 25 °C for the latitudinal and longitudinal samples are presented in Figure 16. Analysing the latitudinal samples, the storage modulus ranges from 3125 to 4000 MPa, the loss modulus ranges from 30 to 80 MPa, and the tan delta averages close to 0.0098 (Figure 16a). For the longitudinal samples, the values for the storage modulus range from 5750 to 7125 MPa, the loss modulus range from 40 to 100 MPa, and the tan delta averages to 0.0082 (Figure 16b). Both types of samples appear to have their lowest tan delta value at very close to 80 Hz. The higher storage modulus of the longitudinal specimens led to a lower tan delta value compared to that of the latitudinal samples; however, the very low tan delta value for both sample types indicates that the carbon-fibre-reinforced PETG demonstrates mostly elastic behaviour, with very little energy dissipation from viscous effects when unloading.

**Figure 17:**
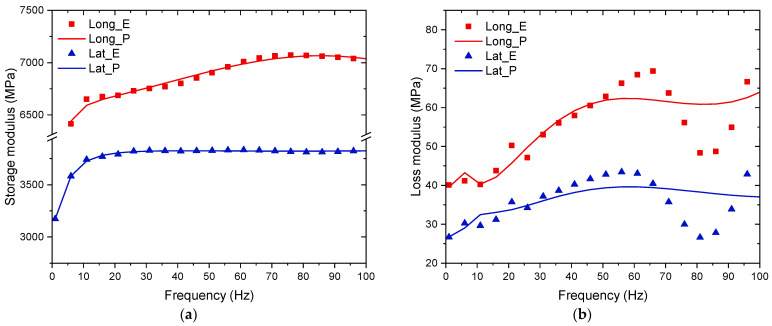
Comparison of storage (**a**) and loss (**b**) moduli from the DMA and their approximation with Prony series (“E” denotes experiments and “P” Prony series).

The Prony series parameters and estimated magnitudes of the shear modulus for each infill pattern are listed in Table 12. The experimental results and the Prony series estimations for the storage and loss moduli over the frequency sweep are compared in Figure 17. The predictions using the Prony series had a very good fit with the experimental values of the storage moduli but a lesser fit with the loss moduli due to their large variation. The algorithm used prioritised the fit of the model for the storage moduli over that for the loss moduli, as this reduced the total residual more (the sum of residuals of all frequencies) for the overall parameters. The parameters outlined in the Prony series calculations can be implemented in finite element models to simulate the viscoelastic response of AM structures and would introduce the damping effect for vibrating structures and dynamic loading, thus offering a more representative analysis of the momentary responses.

## 4. Conclusions

The quasi-static and viscoelastic properties of MEAM CFRPETG were investigated under tension and compression test and a DMA using a frequency sweep. A total of 24 specimen parameters were tested, which involved changing the infill density (40%, 80%, or 100%), the infill pattern (cross-ply, concentric, longitudinal, and latitudinal), and the post-processing conditions (annealed or as-printed). The main conclusions of this study are summarised below:

- The carbon fibres embedded into the polymer matrix act as particulates due to their median length being less than the critical fibre length calculated for this specific combination of polymer and fibres.
- The infill percentage, infill pattern, and annealing had a significant impact on the mechanical properties of the tensile and compressive samples, with the infill percentage being the major contributor in most of the samples, as it directly corresponded to the amount of material in the structure. However, the infill pattern was the major contributor to the variance in the tensile modulus and the yield strain of the compressive specimens.
- Under tensile loads, the longitudinal and concentric specimens demonstrated better performance, showing a significantly greater UTS and tensile modulus than their latitudinal and cross-ply counterparts. The longitudinal annealed samples had UTS, Young’s modulus, and tensile toughness values of 59.4 MPa, 4.58 GPa, and 1.90 MJ m^−3^, respectively. However, annealing did not cause a consistent improvement in the specimens’ performance. This can be attributed to the fact that the samples were annealed for 30 min, which is a short amount of time compared to that used in other studies. Lastly, in terms of toughness (both tensile and compressive), the cross-ply specimens appeared to perform the best out of all infill patterns.
- Under compressive loads, similar observations were made to those under tensile loading, where the longitudinal samples performed the best, followed by the cross-ply samples, which also had the highest toughness. The longitudinal annealed samples had yield stress, Young’s modulus, and compressive toughness values of 68.7 MPa, 1.65 GPa, and 23.76 MJ m^−3^, respectively. Annealing appeared to have a more consistent effect on compressive loads, as the annealed samples enhanced the mechanical properties of the specimens more reliably.
- Overall, the tensile and compressive properties of the longitudinal samples were similar to those of the concentric samples, with certain exceptions. Within tensile testing, the longitudinal samples had a higher UTS and elastic modulus but were more brittle, as evident in their lower strain at failure. Additionally, their toughness was slightly lower than that of other infill patterns, such as cross-ply. In compressive testing, the compressive modulus and yield stress/strain were consistently higher than those of the other infill patterns; however, similar to in the tensile tests, the toughness was greater in other infill patterns.
- Based on findings from the ANOVAs conducted and on the parameters which were changed throughout the investigation, the infill percentage had the greatest contribution to the variance for most of the properties investigated. Some exceptions included the tensile modulus (43.2% contribution of the pattern against 39.9% contribution of the porosity) and the compressive yield strain (57.4% compared to 5.6% porosity contribution). Moreover, it may be advantageous to emphasise the infill pattern of components when a high tensile modulus is required rather than their porosity.
- For certain mechanical properties, some independent variables showed significant interactions between them, i.e., annealing had a greater effect on certain infill types over others. This observation is significant when deciding on the combination of the annealing, infill pattern, and percentage used when a specific mechanical property value is desired. For example, the tensile modulus showed a greater response to annealing in the 80%-infill samples compared to all the other infill percentages.
- Annealing showed promising increases in performance for some samples, especially in terms of the tensile modulus of the specimens. This allows for specific manufacture to obtain certain desirable tensile moduli while also using less material, e.g., if a tensile modulus value of 4.0 GPa is required, an 80%-infill, annealed, longitudinal part may be an ideal substitute for a 100%-infill, as-printed, longitudinal part. This reduces the amount of material used and waste produced, as well as significantly reducing the manufacturing time.
- In the case of the compressive specimens, the annealed parts showed significantly less variance between their values, as was evident by the reduced error in the stress-strain graphs produced.
- Based on the findings from the DMA conducted on the two sample types, it can be concluded that both types of samples exhibit highly elastic behaviour due to their low tan delta values. However, the longitudinal samples exhibited much higher storage moduli throughout the frequency sweep compared to those of the latitudinal samples.

## Figures and Tables

**Table 1:** Printing parameters used for CFRPETG specimens.

Fixed Parameters	Value
Layer height	0.125 mm
Print temperature	245 °C
Material flow rate ^1^	110%
Bed temperature	75 °C
Speed	50 mm/s
Cooling ^2^	20%
Bed adhesion support ^3^	2 mm skirt
Print orientation	Horizontal

^1^ Rate of flow compared to slicer default for specified material. ^2^ Fan speed as a percentage of full fan power. ^3^ Support type for bed adhesion—skirt consists of a single layer height perimeter of 2 mm width.

**Table 2:** Summary of calculated quasi-static tensile mechanical properties of CFRPETG parts (standard deviation in brackets).

Annealing	Infill	Infill Percentage	UTS (MPa)	E (GPa)	Toughness (MJ m^−3^)
As-printed	Concentric	100%	64.3 (2.1)	4.07 (0.08)	21.0 (4.46)
		80%	43.7 (3.5)	2.41 (0.13)	14.0 (2.61)
		40%	11.7 (1.4)	1.11 (0.03)	2.0 (0.71)
	Cross-ply	100%	42.3 (1.0)	1.63 (0.06)	35.3 (12.57)
		80%	38.2 (0.6)	1.44 (0.01)	33.0 (11.38)
		40%	8.9 (0.5)	0.58 (0.01)	4.3 (0.60)
	Latitudinal	100%	33.8 (2.2)	1.34 (0.07)	24.3 (3.07)
		80%	14.3 (1.4)	0.79 (0.05)	6.1 (1.91)
		40%	9.0 (0.4)	0.65 (0.19)	5.9 (1.60)
	Longitudinal	100%	67.8 (2.4)	4.09 (0.04)	33.2 (16.36)
		80%	30.4 (2.5)	2.38 (0.07)	8.1 (1.18)
		40%	23.0 (4.7)	1.13 (0.11)	8.5 (2.52)
Annealed	Concentric	100%	61.7 (4.0)	4.25 (0.25)	23.3 (7.92)
		80%	36.0 (1.9)	3.39 (0.51)	8.0 (0.86)
		40%	15.1 (1.2)	1.08 (0.04)	5.2 (2.00)
	Cross-ply	100%	41 (1.6)	1.72 (0.18)	35.8 (7.80)
		80%	23.9 (0.2)	1.46 (0.23)	19.4 (7.72)
		40%	7.8 (0.4)	0.44 (0.02)	6.4 (3.73)
	Latitudinal	100%	34.3 (2.7)	1.53 (0.14)	31.4 (3.93)
		80%	17.1 (1.6)	1.07 (0.13)	8.1 (2.06)
		40%	6.7 (0.3)	0.45 (0.08)	3.3 (1.22)
	Longitudinal	100%	59.4 (4.1)	4.58 (0.53)	24.2 (5.31)
		80%	37.8 (4.9)	4.13 (0.15)	8.7 (3.95)
		40%	20.0 (2.1)	1.37 (0.14)	5.1 (1.77)

**Table 3:** ANOVA for ultimate tensile strength.

Factor	SS	df	MS	F	P	Contribution
Infill Percentage	17,182.767	2	8591.383	1455.257	0.000	69.25%
Infill Pattern	5246.650	3	1748.883	296.236	0.000	21.14%
Annealing	87.077	1	87.077	14.750	0.000	0.35%
Infill Percentage × Infill Pattern	1451.941	6	241.990	40.990	0.000	5.85%
Infill Percentage × Annealing	19.228	2	9.614	1.628	0.207	0.08%
Infill Pattern × Annealing	84.189	3	28.063	4.753	0.006	0.34%
Infill Percentage × Infill Pattern × Annealing	458.645	6	76.441	12.948	0.000	1.85%
Error	283.377	48	5.904			1.14%
Total	24,813.873	71				100%

SS: sum of squares; df: degrees of freedom; MS: mean squares; F: F-value; P: significance level.

**Table 4:** ANOVA for tensile modulus.

Factor	SS	df	MS	F	P	Contribution
Infill Percentage	51.453	2	25.727	709.620	0.000	39.87%
Infill Pattern	55.677	3	18.559	511.912	0.000	43.15%
Annealing	1.843	1	1.843	50.841	0.000	1.43%
Infill Percentage × Infill Pattern	13.362	6	2.227	61.430	0.000	10.36%
Infill Percentage × Annealing	1.951	2	0.975	26.902	0.000	1.51%
Infill Pattern × Annealing	1.892	3	0.631	17.394	0.000	1.47%
Infill Percentage × Infill Pattern × Annealing	1.122	6	0.187	5.157	0.000	0.87%
Error	1.740	48	0.036			1.35%
Total	129.040	71				100%

**Table 5:** ANOVA for tensile toughness.

Factor	SS	df	MS	F	P	Contribution
Infill Percentage	6841.295	2	3420.648	93.739	0.000	61.26%
Infill Pattern	1148.709	3	382.903	10.493	0.000	10.29%
Annealing	35.280	1	35.280	0.967	0.330	0.32%
Infill Percentage × Infill Pattern	835.561	6	139.260	3.816	0.003	7.48%
Infill Percentage × Annealing	73.026	2	36.513	1.001	0.375	0.65%
Infill Pattern × Annealing	114.621	3	38.207	1.047	0.380	1.03%
Infill Percentage × Infill Pattern × Annealing	368.233	6	61.372	1.682	0.146	3.30%
Error	1751.580	48	36.491			15.68%
Total	11,168.306	71				100%

**Table 6:** Failure types for different CFRPETG tensile specimens.

Failure Type	Failure Location	Samples Within Category		
		Percentage	Annealing	Pattern
Flat (a)	Gauge length (2)	100%	Annealed	Latitudinal
		100%	As-Printed	Cross-ply
		80%	As-Printed	Latitudinal
	Fillet edge (1)	100%	Annealed	Cross-ply
		100%	As-Printed	Latitudinal
		80%	Annealed	Latitudinal
Irregular clean (b)	Gauge length (2)	80%	Annealed	Cross-ply
	Fillet edge (1)	100%	As-Printed	Concentric
		80%	As-Printed	Longitudinal
		40%	Annealed	Longitudinal
		40%	Annealed	Concentric
	Fillet area (3)	100%	Annealed	Longitudinal
		100%	Annealed	Concentric
		100%	As-Printed	Longitudinal
		80%	Annealed	Concentric
Irregular rough (c)	Gauge length (2)	40%	Annealed	Cross-ply
		40%	Annealed	Latitudinal
		40%	As-Printed	Latitudinal
		40%	As-Printed	Cross-ply
	Fillet edge (1)	80%	Annealed	Longitudinal
		80%	As-Printed	Cross-ply
		40%	As-Printed	Concentric
		40%	As-Printed	Longitudinal
	Fillet area (3)	80%	As-Printed	Concentric

**Table 7:** Summary of calculated quasi-static compressive mechanical properties of CFRPETG parts (standard deviation in brackets).

Annealing	Infill	Infill Percentage	E (GPa)	Toughness (MJ m^−3^)	Yield Stress (MPa)	Yield Strain (%)
As-Printed	Concentric	100%	0.53 (0.12)	24.50 (2.74)	22.86 (2.04)	4.50 (0.15)
		80%	0.28 (0.07)	16.07 (1.35)	9.55 (1.34)	3.28 (0.09)
		40%	0.12 (0.04)	4.94 (0.20)	2.36 (0.65)	2.09 (0.04)
	Cross-ply	100%	0.48 (0.11)	31.1 (3.82)	29.24 (3.16)	4.44 (0.15)
		80%	0.36 (0.16)	22.51 (1.43)	10.57 (2.96)	2.61 (0.18)
		40%	0.72 (0.34)	6.58 (0.21)	3.28 (0.83)	3.29 (0.34)
	Latitudinal	100%	0.561 (0.17)	28.63 (1.58)	28.71 (2.90)	4.87 (0.35)
		80%	0.45 (0.08)	20.34 (1.09)	13.92 (1.69)	3.10 (0.24)
		40%	0.13 (0.01)	4.71 (0.35)	4.24 (0.22)	3.46 (0.84)
	Longitudinal	100%	0.80 (0.06)	23.03 (2.67)	58.94 (0.51)	8.97 (1.58)
		80%	0.62 (0.05)	13.28 (1.82)	30.18 (0.78)	5.61 (0.73)
		40%	0.13 (0.02)	2.51 (0.45)	7.31 (3.20)	4.91 (0.64)
Annealed	Concentric	100%	0.60 (0.04)	29.76 (0.56)	35.65 (0.20)	4.97 (0.05)
		80%	0.26 (0.07)	15.27 (0.73)	10.95 (0.65)	4.18 (0.17)
		40%	0.097 (0.02)	4.76 (0.94)	3.21 (0.32)	3.43 (0.08)
	Cross-ply	100%	1.07 (0.08)	34.78 (0.76)	38.94 (0.53)	5.10 (0.84)
		80%	0.38 (0.03)	21.13 (0.37)	10.91 (0.26)	2.91 (0.11)
		40%	0.094 (0.02)	4.83 (0.45)	2.67 (0.23)	2.95 (0.05)
	Latitudinal	100%	0.91 (0.06)	29.86 (1.34)	32.85 (0.84)	3.84 (0.12)
		80%	0.56 (0.11)	18.57 (2.10)	13.13 (0.11)	3.28 (0.05)
		40%	0.13 (0.02)	3.76 (0.74)	4.12 (0.13)	3.18 (0.03)
	Longitudinal	100%	1.65 (0.11)	23.76 (0.97)	68.70 (2.00)	7.35 (0.21)
		80%	0.76 (0.05)	15.17 (0.51)	37.07 (3.24)	6.06 (1.01)
		40%	0.19 (0.02)	2.26 (0.26)	9.22 (1.58)	5.56 (0.35)

**Table 8:** ANOVA for compressive yield strength.

Factor	SS	df	MS	F	P	Contribution
Infill Percentage	12,745.788	2	6372.894	418.078	0.000	57.86%
Infill Pattern	5721.861	3	1907.287	125.123	0.000	25.97%
Annealing	162.162	1	162.162	10.638	0.002	0.74%
Infill Percentage × Infill Pattern	2394.910	6	399.152	26.185	0.000	10.87%
Infill Percentage × Annealing	94.505	2	47.253	3.100	0.054	0.43%
Infill Pattern × Annealing	104.764	3	34.921	2.291	0.090	0.48%
Infill Percentage × Infill Pattern × Annealing	73.226	6	12.204	0.801	0.574	0.33%
Error	731.680	48	15.243			3.32%
Total	22,028.897	71				100%

**Table 9:** ANOVA for compressive yield strain.

Factor	SS	df	MS	F	P	Contribution
Infill Percentage	11.570	2	5.785	5.336	0.008	5.46%
Infill Pattern	121.747	3	40.582	37.436	0.000	57.41%
Annealing	1.717	1	1.717	1.584	0.214	0.81%
Infill Percentage × Infill Pattern	18.238	6	3.040	2.804	0.020	8.60%
Infill Percentage × Annealing	3.510	2	1.755	1.619	0.209	1.66%
Infill Pattern × Annealing	1.402	3	0.467	0.431	0.732	0.66%
Infill Percentage × Infill Pattern × Annealing	1.863	6	0.311	0.286	0.941	0.88%
Error	52.034	48	1.084			24.54%
Total	212.082	71				100%

**Table 10:** ANOVA for compressive modulus.

Factor	SS	df	MS	F	P	Contribution
Infill Percentage	8,522,335	2	4,261,167.654	258.999	0.000	70.78%
Infill Pattern	1,189,179	3	396,393.267	24.093	0.000	9.88%
Annealing	240,518	1	240,518.482	14.619	0.000	2.00%
Infill Percentage × Infill Pattern	437,252	6	72,875.420	4.429	0.001	3.63%
Infill Percentage × Annealing	594,878	2	297,439.042	18.079	0.000	4.94%
Infill Pattern × Annealing	90,110	3	30,036.828	1.826	0.155	0.75%
Infill Percentage × Infill Pattern × Annealing	177,368	6	29,561.441	1.797	0.120	1.47%
Error	789,716	48	16,452.417			6.56%
Total	12,041,359	71				100%

**Table 11:** ANOVA for compressive toughness.

Factor	SS	df	MS	F	P	Contribution
Infill Percentage	8190.419	2	4095.209	1888.288	0.000	91.55%
Infill Pattern	401.682	3	133.894	61.738	0.000	4.49%
Annealing	14.334	1	14.334	6.609	0.013	0.16%
Infill Percentage × Infill Pattern	160.557	6	26.759	12.339	0.000	1.79%
Infill Percentage × Annealing	39.588	2	19.794	9.127	0.000	0.44%
Infill Pattern × Annealing	9.804	3	3.268	1.507	0.225	0.11%
Infill Percentage × Infill Pattern × Annealing	26.370	6	4.395	2.027	0.080	0.29%
Error	104.100	48	2.169			1.16%
Total	8946.853	71				100%

**Table 12:** Prony series parameters for MEAM CFRPETG structures.

Infill Pattern	$G_{0}$	i	1	2	3	4	5	6	7
Longitudinal	1573	$G_{i}$	−6.2348	4.4381	−8.6978	4.5598	0.2818	7.2684	0.3236
		$\tau_{i}$	−0.0050	−0.0066	0.0060	339.7521	−0.3149	0.0071	0.3092
Latitudinal	515	$G_{i}$	−6.6890	−0.4546	0.6753	−214.9619	0.4821	0.5321	7.0823
		$\tau_{i}$	−0.0002	−0.0101	−0.0062	147.5579	−0.1762	0.1599	4.4789

## Data Availability

The original contributions presented in the study are included in the article, further inquiries can be directed to the corresponding author.
